# Fed up: a qualitative investigation on the influencing factors of food insecurity in the Azores

**DOI:** 10.1093/eurpub/ckac130.105

**Published:** 2022-10-25

**Authors:** R Freiheit, T Leão

**Affiliations:** University of Porto, Institute of Public Health, Porto, Portugal

## Abstract

**Background:**

In 2019, over 1 billion people experienced food insecurity. In the Azores, a 9-island archipelago in the Atlantic Ocean, 13.4% of its population perceived food insecurity, the highest regional rate in Portugal (10.1%). These geographical limitations paired with high unemployment and low education rates, may challenge their access to nutritious food. It is still unclear how these (and other) contextual factors may affect access and availability to food in this remote area, and how they interact with individual determinants.

**Methods:**

Data collection was conducted in early 2022 with 13, in-depth, 1:1 semi-structured interviews regarding topics of food consumption and supply in the Azores. Non-probability sampling was used, with use of purposive and snowball sampling. We included key-stakeholders currently living in the islands or working in a role related to the islands (i.e., in healthcare, supply chain, agriculture, etc.). We performed a thematic analysis with an inductive-deductive iterative analysis.

**Results:**

Participants identified several challenges related to access and availability of food. Vegetables, fruit and fish can be scarce and are subject to seasonal variation. There are clear regional disparities in access to food, with more isolated islands having less variety, quality and quantity of healthy foods available. Its topography and weather limit agricultural practices and transportation, but education, culture, and policies subsidizing the production of meat and dairy were also identified as important barriers to local production of fruits and vegetables.

**Conclusions:**

Food insecurity is a public health concern in this outermost EU region. If topography and geography limit the production and access of nutritious foods, economic and agricultural policies are a leading theme of concern to highlight. The solution for food insecurity thus demands an interdisciplinary dialogue to better adjust future policies in the Azores and in similar regions.

**Key messages:**

- Food insecurity is a public health issue in the Azores, a European outermost region. Access to nutritious foods is unstable due to contextual variables such as geography, weather and policy.
- A possible solution to address food insecurity in the Azores islands demands interdisciplinary dialogue to better adjust future policies in this region and within similar contexts.

